# New Medical College

**Published:** 1882-01

**Authors:** 


### Editorial.

### ARTICLE XIII.

THE preliminary announcement of the Medical College, lately organized in this city, has been laid before the members of the profession. Its published announcement of its proposed methods of securing competent medical instructors has also in these pages been given to the world. The ground for the new building has been purchased; and, in brief, it may be said, that the college is practically before the profession and the public to be judged upon its merits.

However much the supposed necessity for the multiplication of new schools in medicine may be deplored, it is certainly gratifying to note that each, as it comes forward, acknowledges the imperative necessity of announcing a broad advance in the standards and requirements for its matriculates and forthcoming graduates. The Chicago College of Physicians is no exception to the rule. The Board of Directors declare that the institution is "formed in the interests of a higher and more thorough medical education; and that they intend to make its organization, equipment and plans of teaching so excellent and complete in every particular, as to enable it to take rank with the foremost medical schools in this country. The highest practical standard of preliminary acquirement will be fixed and exacted; a pupilage of at least four years, including a graded course of instruction covering at least three full winter sessions, will be compulsory; and the most satisfactory proofs of proficiency in every department of medical knowledge will be required of all candidates for graduation. The examinations for a degree will be conducted by a committee of examiners separate and distinct from its teaching corps."

Certainly this is a high and worthy standard; and if it shall be rigidly maintained and successfully applied, not only the medical men of this State, but the profession throughout the Northwest, will have reason to be thankful that the new medical school was called into existence. Such an institution needs no excuse for its existence. It is itself the best explanation and apology for its perpetuity. Its *raison d'être* is the survival of the fittest.

---

### BOOKS AND PAMPHLETS RECEIVED.

Manual of Midwifery. By Alfred Meadows. Fourth Edition. G. P. Putnam's Sons.

Diseases of the Eye. By Henry D. Noyes, M.D. Wm. Wood & Co. Medical Library, 1881.

Handbook of Uterine Therapeutics. By E. J. Tilt, M.D., London. Wood's Library, 1881.

A Study on Tumors of the Bladder. By Alex. W. Stein, New York. Wm. Wood & Co.

Frozen Sections of a Child. By Thomas Dwight, M.D. Wm. Wood & Co.

Suppression of Urine. By E. P. Fowler, M.D. Wm. Wood & Co., 1881.

Lectures on Electricity. By A. D. Rockwell, M.D. Wm. Wood & Co., N. Y.

Pocket Book of Physical Diagnosis. By Dr. Edward T. Broen. Blakiston, Philadelphia.

---

THESE are the characters by which you are to recognize a hernia of the epiploon alone: The tumor is dull, and presents no gurgling on pressure; you will find these signs described in your books, and they are deceptive, for an entero-epiplocele presents these characters; but one symptom, to which I most especially direct your attention, is the narrowness of the pedicle of the hernia, and the almost complete indolence of this on pressure, joined to the absence of a resistant plane behind the ring. This narrowness of the neck is explained without difficulty, when we recall the texture of the epiploon. We understand very easily that the fat may be depressed by the constricting band, as by a thread.—*M. Desres, in Gazette des Hôpitaux.*

